# Current hotspot and study trend of innate immunity in COVID-19: a bibliometric analysis from 2020 to 2022

**DOI:** 10.3389/fimmu.2023.1135334

**Published:** 2023-05-10

**Authors:** Ping Lai, Shuquan Xu, Jin-hua Xue, Hong-zhou Zhang, Yi-ming Zhong, Yong-ling Liao

**Affiliations:** ^1^ Department of Cardiology, First Affiliated Hospital of Gannan Medical University, Gannan Medical University, Ganzhou, China; ^2^ Key Laboratory of Prevention and Treatment of Cardiovascular and Cerebrovascular Diseases, Ministry of Education, Gannan Medical University, Ganzhou, China; ^3^ Institute of Experimental Cardiovascular Research, University Medical Center Hamburg-Eppendorf, Hamburg, Germany; ^4^ German Center for Cardiovascular Research (DZHK), Partner Site Hamburg/Kiel/Lübeck, Hamburg, Germany; ^5^ The First School of Clinical Medicine, Gannan Medical University, Ganzhou, China; ^6^ School of Basic Medicine, Gannan Medical University, Ganzhou, China

**Keywords:** COVID-19, innate immunity, bibliometric analysis, WoSCC, VOS viewer, CiteSpace

## Abstract

**Background:**

Since the coronavirus disease 2019 (COVID-19) has spread throughout the world, many studies on innate immunity in COVID-19 have been published, and great progress has been achieved, while bibliometric analysis on hotspots and research trends in this field remains lacking.

**Methods:**

On 17 November 2022, articles and reviews on innate immunity in COVID-19 were recruited from the Web of Science Core Collection (WoSCC) database after papers irrelevant to COVID-19 were further excluded. The number of annual publications and the average citations per paper were analyzed by Microsoft Excel. Bibliometric analysis and visualization of the most prolific contributors and hotspots in the field were performed by VOSviewer and CiteSpace software.

**Results:**

There were 1,280 publications that met the search strategy on innate immunity in COVID-19 and were published from 1 January 2020 to 31 October 2022. Nine hundred thirteen articles and reviews were included in the final analysis. The USA had the highest number of publications (Np) at 276 and number of citations without self-citations (Nc) at 7,085, as well as an H-index of 42, which contributed 30.23% of the total publications, followed by China (Np: 135, Nc: 4,798, and H-index: 23) with 14.79% contribution. Regarding Np for authors, Netea, Mihai G. (Np: 7) from the Netherlands was the most productive author, followed by Joosten, Leo A. B. (Np: 6) and Lu, Kuo-Cheng (Np: 6). The Udice French Research Universities had the most publications (Np: 31, Nc: 2,071, H-index: 13), with an average citation number (ACN) at 67. The journal *Frontiers in Immunology* possessed the most publications (Np: 89, Nc: 1,097, ACN: 12.52). “Evasion” (strength 1.76, 2021-2022), “neutralizing antibody” (strength 1.76, 2021-2022), “messenger RNA” (strength 1.76, 2021-2022), “mitochondrial DNA” (strength 1.51, 2021-2022), “respiratory infection” (strength 1.51, 2021-2022), and “toll-like receptors” (strength 1.51, 2021-2022) were the emerging keywords in this field.

**Conclusion:**

The study on innate immunity in COVID-19 is a hot topic. The USA was the most productive and influential country in this field, followed by China. The journal with the most publications was *Frontiers in Immunology*. “Messenger RNA,” “mitochondrial DNA,” and “toll-like receptors” are the current hotspots and potential targets in future research.

## Introduction

Severe acute respiratory syndrome coronavirus 2 (SARS-CoV-2) caused an emergency disease pandemic worldwide, named coronavirus disease 2019 (COVID-19), which led to a considerable threat to public health and economic development (1). SARS-CoV-2 is a positive-sense single-stranded RNA (ssRNA) virus that belongs to the genus *Betacoronavirus*. It owns six typical functional open reading frames (ORFs), namely, replicase (ORF1a/ORF1b), spike (S), envelope (E), membrane (M), and nucleocapsid (N), which are shared by other betacoronaviruses (2). Notably, the distribution and replication of SARS-Cov-2 were found in the respiratory system and the genitourinary, gastrointestinal, and even central neural systems (3). Therefore, SARS-CoV-2 can be detected in urine and stool, except in samples from the respiratory tract, which increased the risk of infection *via* sewage networks and wastewater systems (4).

The transmissibility of viruses was increasing with the evolution of new variants. The primary reproduction number (*R*_0_) indicates the average number of patients who would be infected by a primary case in a totally susceptible population, elevating from the original SARS-CoV-2 (*R*_0_: 2-2.5) to Delta (*R*_0_: 3.2-8). Currently, the Omicron variant, which has sparked a wave of infections worldwide, has an *R*_0_ value 3.2 times higher than the Delta variant (5, 6). Various vaccines were produced to prevent the spread of SARS-CoV-2. However, as an ssRNA virus, SARS-CoV-2 reduces the efficiency of vaccines with high-frequency mutation. Vaccine breakthrough has been observed in different variants, such as Gamma (P.1), Delta (B.1.617.2), and Omicron (B.1.1.529) (7–9). It is no longer realistic to resist SARS-CoV-2 with vaccines alone, but support from research on innate immunity aspects is required.

Growing studies have clarified that innate immunity plays a key role in SARS-CoV-2 infection. After SARS-CoV-2 entered the targeting cells, virion or viral RNA was detected by cGAS/STING or melanoma differentiation-associated gene 5 (MDA-5) or both, leading to the release of type I/III interferon by activating interferon regulatory factor 3 (IRF3) and the nuclear factor-kappa B (NF-kB) pathway. Otherwise, SARS-CoV-2 infected permissible cells *via* angiotensin-converting enzyme 2 (ACE2) and was taken up by the endosome. Furthermore, viruses were recognized by toll-like receptors (TLRs) 7/9 or TRL 3, which induce the secretion of massive inflammatory cytokines *via* activating the NF-kB pathway (10).

Various cytokines, particularly interleukin-1b, interleukin-6 (IL-6), and tumor necrosis factor-α, were elevated in mild and severe COVID-19 patients (11). Cytokine storm (CS) was considered a major concern in acute respiratory distress syndrome (ARDS), multiorgan failure, and even death among COVID-19 patients (12). A sharply increased level of cytokines always induces CS, mainly caused by innate immune cells, despite both innate and adaptive immunity inducing CS (13, 14). Among the elevated cytokines in COVID-19 patients’ sera, IL-6 has been proven to be associated with CS (15). Thus, tocilizumab, a monoclonal IL-6R antibody that blocks the IL-6 signaling pathway and potentially reduces the risk of CS, was a recommended therapy for COVID-19. In addition, more medicines targeting innate immunity were recommended, such as Janus kinase inhibitors, RNA-dependent RNA polymerase (RdRp) inhibitors, and soluble ACE2 fused with the immunoglobulin Fc domain (ACE2-Fc). However, the efficacy is unclear and more investigation is necessary (16). Taken together, innate immunity was crucial to the emergence of COVID-19 and may offer a potential treatment.

Bibliometric analysis offered an intuitive and effective foundation for integrating information, reflected interconnections between them, and predicted the development of cutting-edge advances in the area based on publications in this field (17, 18). Recently, bibliometric analysis was widely adopted by scholars in neurology (19), vaccination (20), and otorhinolaryngology (21) related to COVID-19, especially the bibliometric analysis based on VOSviewer and CiteSpace software (22, 23). However, bibliometric analysis of innate immunity in COVID-19 remains lacking. Therefore, this study aims to show the progress, hotspots, and frontiers in the field based on the published literature and provide a strong foundation for future research.

## Material and methods

### Source *database and data c*ollection

**Figure 1:**
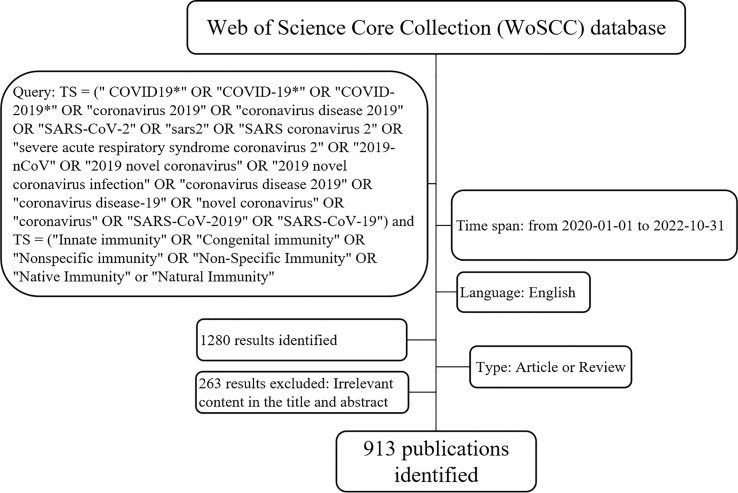
Flowchart for choosing papers in the current study.

The Web of Science Core Collection (WoSCC) database was chosen as the data source since it is a comprehensive, scientific publishing research database widely used by researchers (24). The search formula was as follows: TS (Topics) = (“COVID19*” OR “COVID-19*” OR “COVID-2019*” OR “coronavirus 2019” OR “coronavirus disease 2019” OR “SARS-CoV-2” OR “sars2” OR “SARS coronavirus 2” OR “severe acute respiratory syndrome coronavirus 2” OR “2019-nCoV” OR “2019 novel coronavirus” OR “2019 novel coronavirus infection” OR “coronavirus disease 2019” OR “coronavirus disease-19” OR “novel coronavirus” OR “coronavirus” OR “SARS-CoV-2019” OR “SARS-CoV-19”) and TS = (“Innate immunity” OR “Congenital immunity” OR “Nonspecific immunity” OR “Non-Specific Immunity” OR “Native Immunity” or “Natural Immunity”). The publication language was restricted to English, the published time was set from 1 January 2020 to 31 October 2022, only articles or reviews were retrieved, and all data were gathered on 17 November 2022. Papers irrelevant to COVID-19 were further excluded *via* manual screening by two authors (Shuquan Xu, Jin-hua Xue) independently, and any controversy was finally decided by Yong-ling Liao and Ping Lai. The specific flowchart is shown in **Figure 1**. Additionally, the 2021 impact factor (IF), the 2021 journal citation report (JCR), and the Hirsch index (H-index) were extracted directly from the WoSCC.

### Data analysis and visualization

Microsoft Excel 2019 processed the data and analyzed the annual distribution of publications and average citations per publication. VOSviewer (version 1.6.18) (link: https://www.vosviewer.com/download), developed by Nees Jan van Eck and Ludo Waltman (25), was used to identify productive countries, authors, and most cited publications based on bibliographic data.

CiteSpace (version 6.1.R3) (link: https://sourceforge.net/projects/citespace/files/) developed by Chaomei Chen (26) was employed for detecting clusters of keywords from publications with high citation bursts, and creating the visual map of the keyword network from the timeline view was completed by CiteSpace. The included CiteSpace parameters were as follows: period (2020–2022), years per slice (one year), term source (title, abstract, author keyword, keyword plus), node types (keyword), links (strength: cosine, scope: within slices), selection criteria (g-index: *k* = 25), and pruning (minimum spanning tree, pruning sliced networks, pruning the merged network). The logarithmic likelihood rate was used as the clustering algorithm, and all clusters were labeled with keywords.

## Results

### Temporal distribution and description of *p*ublications

**Figure 2:**
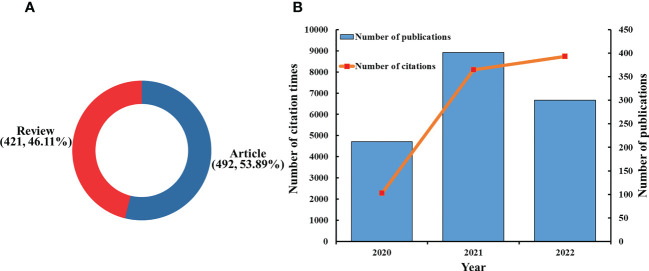
**(A)** Distribution of publications by type. **(B)** The number of annual publications and citations of innate immunity in COVID-19 from 2020 to 2022.

There were 1,280 articles that met the search formula, comprising 683 articles, 493 reviews, 32 meeting abstracts, 55 editorial materials, and 17 early access articles (**Figure 1**). A total of 913 publications consisting of 421 reviews (46.11%) and 492 articles (53.89%) were included in the final analysis (**Figure 2A**). The distribution of the annual number of publications and total citations over time on innate immunity in COVID-19 **is** shown in **Figure 2B**. The number of publications (Np) increased from 212 in 2020 to 401 in 2021, and 300 papers were published in the first 10 months of 2022. The total number of citations (Nc) was 19,138, the average citation of publications was high at 20.96, and the H-index was 60.

### Country and region distribution

**Figure 3:**
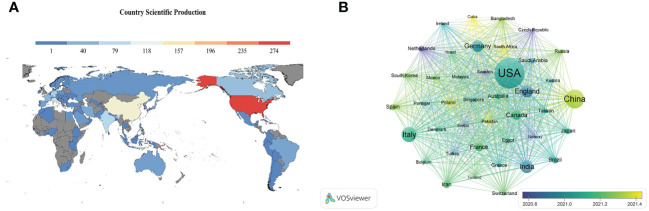
**(A)** Geographical distribution map of global publications related to innate immunity in COVID-19. Colors ranging from cold to warm represent an increasing number of publications. **(B)** Visual network of a country or region with more than five publications. Each network node represents a different country or region; the larger the node indicates the more publications. The thicker the line linking the nodes reflects the closer the cooperation between the countries or regions.

A total of 87 countries or regions contributed to all publications. The geographical distribution map showed that most publications on innate immunity in COVID-19 were from North America, Asia, and Europe (**Figure 3A**). **Table 1** illustrates the top 10 fruitful countries or regions. The leading country was the USA (Np: 276, Nc: 7,085, H-index: 42) with 30.23% of the total publications, followed by China (Np: 135, Nc: 4,798, H-index: 23) and Italy (Np: 97, Nc: 1,438, H-index: 21), with 14.79% and 10.62%, respectively. Among all countries or regions, 41 countries or regions had more than five publications (**Figure 3B**). Among these prolific countries or regions, the USA had the most collaboration with other countries.

**Table 1:** The top 10 countries or regions related to innate immunity in COVID-19.

Rank	Country	Np	Nc	H-index	ACN
1	USA	276	7,085	42	26.19
2	China	135	4,798	23	36.32
3	Italy	97	1,438	21	15.09
4	India	76	1,256	16	16.62
5	England	70	2,575	20	36.61
6	Germany	67	3,310	22	49.79
7	Canada	51	833	15	16.47
8	France	43	2,128	14	49.67
9	Iran	32	277	9	8.78
10	Brazil	29	342	10	11.86

Np, number of publications; Nc, number of citations without self-citations; ACN, average citation number.

### Analysis of authors and institutions

**Figure 4:**
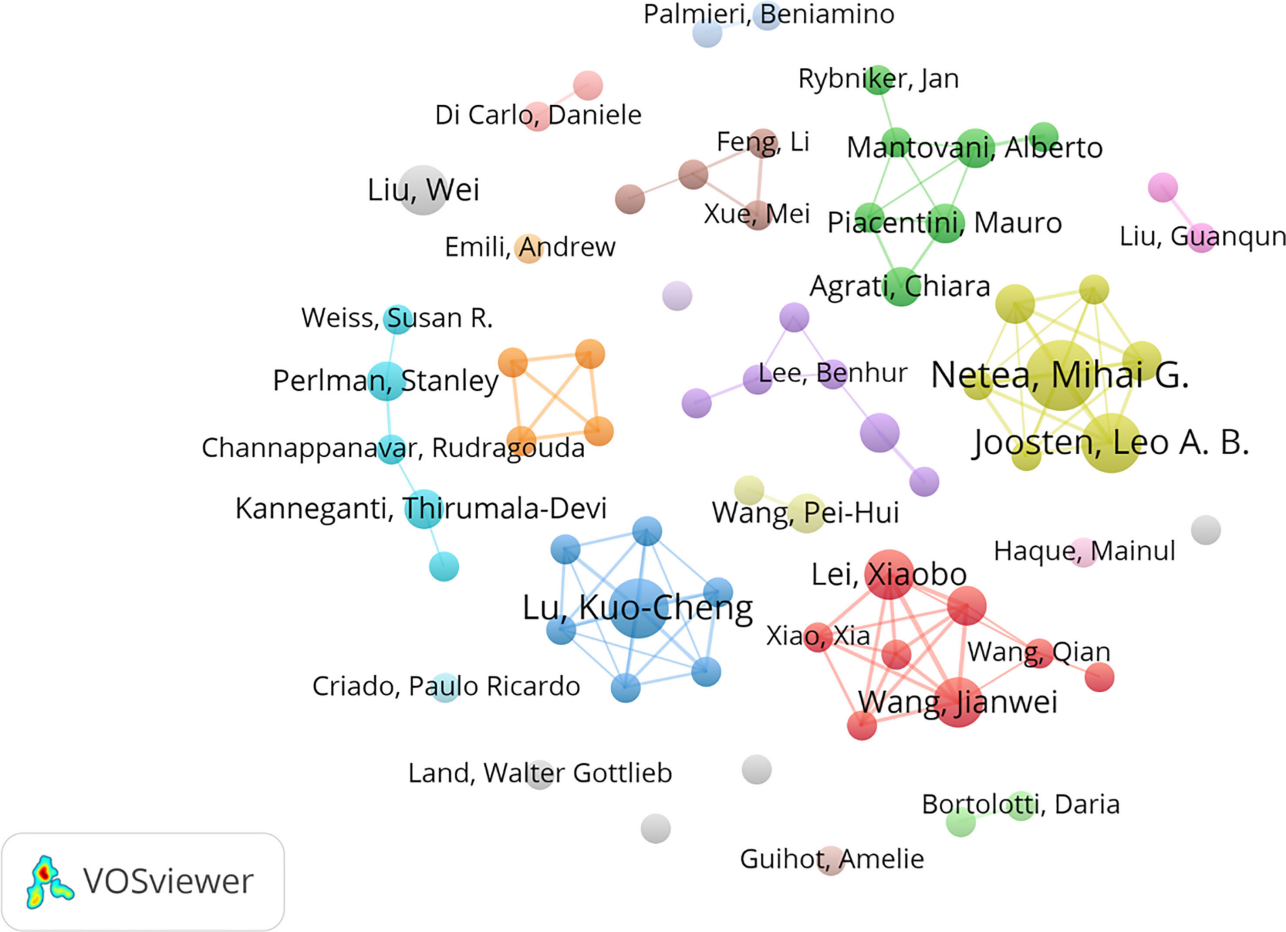
Visual network map of 68 authors with more than three papers. The node size stands for the number of publications. Lines between nodes represent cooperation between authors.

A total of 5,836 authors from 1,869 institutions contributed to all publications. **Table 2** lists the top 10 most prolific authors on innate immunity in COVID-19. The authors with more than three publications are shown in **Figure 4**. The most productive author was Netea, Mihai G. (Np: 7), who was from the Netherlands, followed by Joosten, Leo A. B. (Np: 6) and Lu, Kuo-Cheng (Np: 6). Notably, the prolific authors were mainly from China, the Netherlands, and the USA.

**Table 2:** The top 10 most prolific authors and institution of innate immunity in COVID-19.

Rank	Author	Np	Total citations	ACN	Country	
1	Netea, Mihai G.	7	178	24.3	Netherlands	
2	Joosten, Leo A. B.	6	171	27.8	Netherlands	
3	Lu, Kuo-Cheng	6	64	10.7	China	
4	Lei, Xiaobo	5	506	101.2	China	
5	Wang, Jianwei	5	506	101.2	China	
6	Liu, Wei	5	37	7.4	China	
7	Ren, Lili	4	504	126.0	China	
8	Kanneganti, Thirumala-Devi	4	364	91.0	USA	
9	Wang, Pei-Hui	4	143	35.8	China	
10	Perlman, Stanley	4	124	31.0	USA	
	**Institution**					
1	Udice French Research Universities	31	2,071	67.0	France	
2	University of California System	28	505	18.4	USA	
3	Institute national de la santé et de la recherche medicale	27	641	23.9	France	
4	Chinese Academy of Sciences	21	702	33. 7	China	
5	University of London	19	1,773	93.5	UK	
6	Harvard University	18	2,161	120.2	USA	
7	Centre national de la recherche scientifique	17	1,760	103.6	France	
8	University of Texas System	17	347	20.5	USA	
9	National Institutes of Health	16	221	13.8	USA	
10	US Department of Veterans Affairs	16	251	15.8	USA	

Np, number of publications; Nc, number of citations without self-citations; ACN, average citation number.

The top 10 institutions ranked by Np are listed in **Table 2**. Five institutions with the most publications were located in the USA, three in France, one in China, and one in the UK. Udice French Research Universities [Np: 31, Nc: 2,071, H-index: 13, average citation number (ACN): 67] from France was the most productive institution, followed by the University of California System (Np: 28, Nc: 505, H-index: 10, ACN: 18.43) from the USA and Institute national de la santé et de la recherche medicale (Np: 27, Nc: 641, H-index: 10, ACN: 23.96) from France.

### Distribution of journals and disciplines

The top 10 journals with the highest number of publications (**Table 3**) contributed 26.6% of all publications in this field. Among the top 10 journals, *Frontiers in Immunology* had the most publications (Np: 89), followed by the *International Journal of Molecular Sciences* (Np: 34), *Viruses-Basel* (Np: 28), *Cells* (Np: 17), and *Frontiers in Microbiology* (Np: 14). However, in terms of the ACN, the *Journal of Medical Virology* ranked first (ACN: 21.92), followed by the *Journal of Virology* (ACN: 19.86) and *Medical Hypotheses* (ACN: 13.82). Six of the top 10 journals belong to the JCR Q1 area. Specifically, the journal with the highest impact factor is *Journal of Medical Virology* (IF: 20.693), followed by *Frontiers in Immunology* (IF: 8.787). Additionally, the top 10 disciplines in this field were led by immunology (Np: 253), biochemistry molecular biology (Np: 144), and cell biology (Np: 96). Meanwhile, immunology (Nc: 4,030, H-index: 44) had the highest H-index and Nc (**Table 4**).

**Table 3:** The top 10 active institutions and journals of innate immunity in COVID-19.

Rank	Journal	Np	Nc	ACN	IF	JCR
1	*Frontiers in Immunology*	89	1,097	12.52	8.787	Q1
2	*International Journal of Molecular Sciences*	34	316	9.41	6.208	Q1
3	*Viruses-Basel*	28	162	5.86	5.818	Q2
4	*Cells*	17	204	12.00	7.666	Q2
5	*Frontiers in Microbiology*	14	43	3.07	6.064	Q1
6	*Journal of Virology*	14	276	19.86	6.549	Q2
7	*Journal of Medical Virology*	13	284	21.92	20.693	Q1
8	*Frontiers in Cellular and Infection Microbiology*	12	33	2.75	6.073	Q1
9	*mBio*	11	121	11.00	7.786	Q1
10	*Medical Hypotheses*	11	152	13.82	4.411	Q2

Np, number of publications; Nc, number of citations without self-citations; ACN, average citation number; IF, impact factor; JCR, journal citation report.

**Table 4:** The top 10 subject categories.

Rank	WOS categories	Np	Nc	H-index
1	Immunology	253	4,030	37
2	Biochemistry molecular biology	144	4,292	23
3	Cell biology	96	4,030	23
4	Microbiology	96	1,086	19
5	Virology	89	1,277	18
6	Medicine research experimental	86	2,613	18
7	Pharmacology pharmacy	67	901	16
8	Chemistry multidisciplinary	47	409	12
9	Multidisciplinary sciences	39	1,934	16
10	Infectious diseases	31	517	13

Np, number of publications; Nc, number of citations without self-citations.

### Highly cited literature analysis

**Figure 5:**
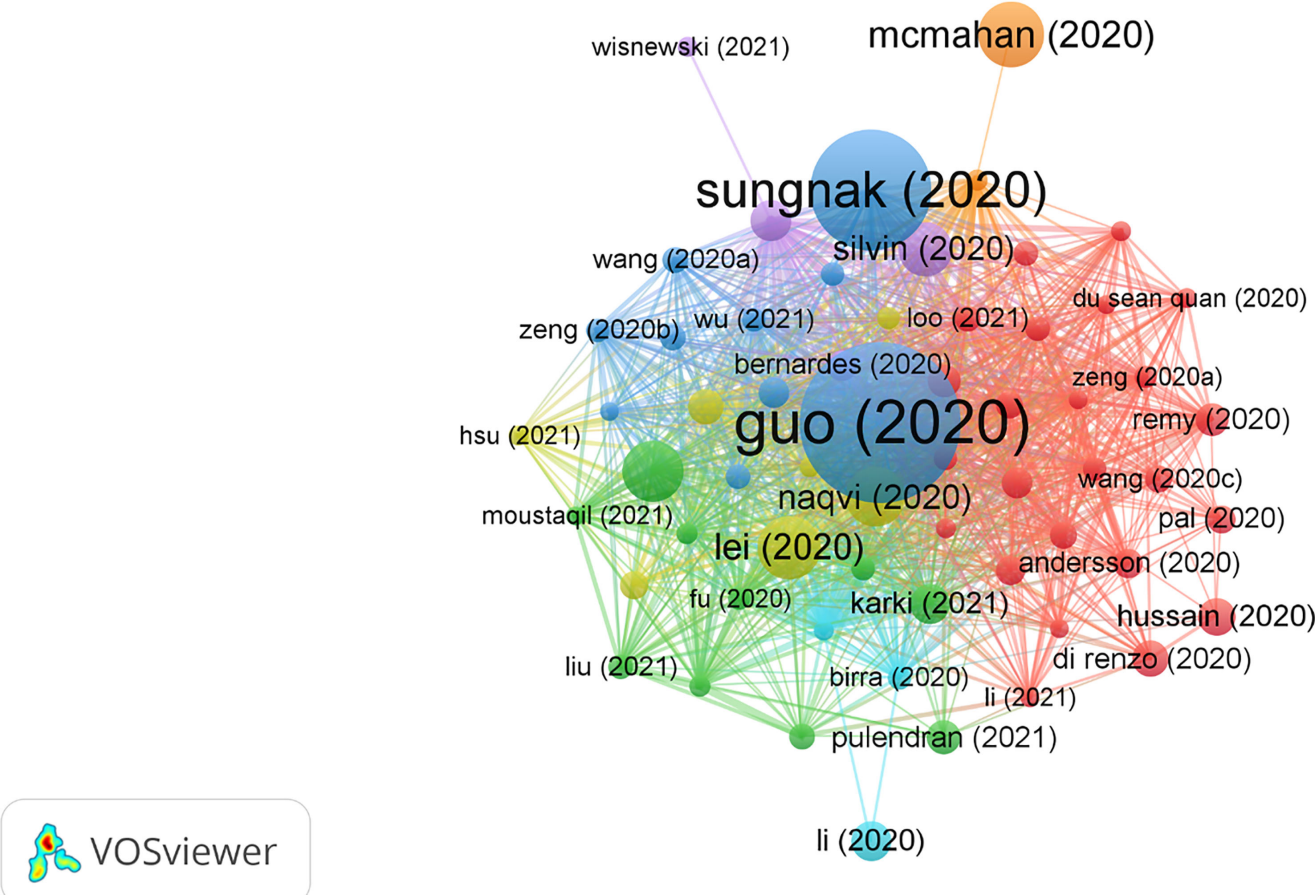
Visual analysis of 60 publications with more than 60 citations in this field. Different colors indicate different themes of the publications, and the node size denotes the number of citations. Lines between nodes stand for relevance between various publications.

The top 10 most cited publications are illustrated in **Table 5**. Among those 10 publications, seven were articles and three were reviews. There were 60 publications with Nc more than 60 times (**Figure 5**).

**Table 5:** The top cited publications.

Publications	Journal	Type	Authors	Year	Citations
The origin, transmission and clinical therapies on coronavirus disease 2019 (COVID-19) outbreak - an update on the status	*Military Medical Research*	Review	Guo, Yan-Rong et al.	2020	2,121
SARS-CoV-2 entry factors are highly expressed in nasal epithelial cells together with innate immune genes	*Nature Medicine*	Article	Sungnak, Waradon et al.	2020	1,323
Activation and evasion of type I interferon responses by SARS-CoV-2	*Nature Communications*	Article	Lei, Xiaobo et al.	2020	454
Papain-like protease regulates SARS-CoV-2 viral spread and innate immunity	*Nature*	Article	Shin, Donghyuk et al.	2020	425
Insights into SARS-CoV-2 genome, structure, evolution, pathogenesis and therapies: Structural genomics approach	*Biochimica et Biophysica Acta-Molecular Basis of Disease*	Review	Naqvi, Ahmad Abu Turab et al.	2020	415
Elevated Calprotectin and Abnormal Myeloid Cell Subsets Discriminate Severe from Mild COVID-19	*Cell*	Article	Silvin, Aymeric et al.	2020	345
COVID-19 and the human innate immune system	*Cell*	Review	Schultze, Joachim L et al.	2021	219
The ORF6, ORF8 and nucleocapsid proteins of SARS-CoV-2 inhibit type I interferon signaling pathway	*Virus Research*	Article	Li, Jin-Yan et al.	2020	209
Synergism of TNF-alpha and IFN-gamma Triggers Inflammatory Cell Death, Tissue Damage, and Mortality in SARS-CoV-2 Infection and Cytokine Shock Syndromes	*Cell*	Article	Karki, Rajendra et al.	2021	207
COVID-19: Is there a role for immunonutrition in obese patient?	*Journal of Translational Medicine*	Review	Di Renzo, Laura et al.	2020	178

In 2020, Guo, Yan-Rong et al. published a review paper titled “The origin, transmission, and clinical therapies on coronavirus disease 2019 (COVID-19) outbreak - an update on the status” in *Military Medical Research* (27), with 2,121 citations, which was at the top of the list. This article summarized the latest research progress on the epidemiology, pathogenesis, and clinical characteristics of COVID-19 and discussed current treatment and scientific advances to combat the novel coronavirus epidemic.

The title of the second most cited publication is “SARS-CoV-2 entry factors are highly expressed in nasal epithelial cells together with innate immune genes,” which was published in *Nature Medicine* in 2020 by Waradon Sungnak et al. (28). This paper indicated that viral entry-associated genes are co-expressed in nasal epithelial cells with genes involved in innate immunity, which highlights the potential role of cells in initial viral infection, spread, and clearance. The study offers a helpful resource for further research lines with valuable clinical samples from COVID-19 patients.

The third most cited paper is titled “Activation and evasion of type I interferon responses by SARS-CoV-2,” published by Lei, Xiaobo, et al. in *Nature Communication* (29). This study showed that SARS-CoV-2 perturbs the host innate immune response *via* its structural and non-structural proteins and thus provides insights into the pathogenesis of SARS-CoV-2.

### Evolution of keywords

Keywords were extracted from all 913 publications for co-occurrence analysis by CiteSpace. Among the top 20 high-frequency keywords, “innate immunity,” “infection,” “activation,” “coronavirus,” and “expression” ranked first to fifth with a frequency of 453, 126, 118, 99, and 90, respectively (**Table 6**).

**Table 6:** The top 20 keywords in the publications.

Rank	Keyword	Frequency	Rank	Keyword	Frequency
**1**	innate immunity	453	**11**	cytokine storm	64
**2**	infection	126	**12**	respiratory syndrome coronavirus	63
**3**	activation	118	**13**	sar	61
**4**	coronavirus	99	**14**	virus	58
**5**	expression	90	**15**	response	57
**6**	cell	85	**16**	nf kappa b	54
**7**	immune response	79	**17**	sars coronavirus	54
**8**	covid 19	79	**18**	I interferon	52
**9**	receptor	69	**19**	RIG-I	47
**10**	protein	66	**20**	dendritic cell	46

**Figure 6:**
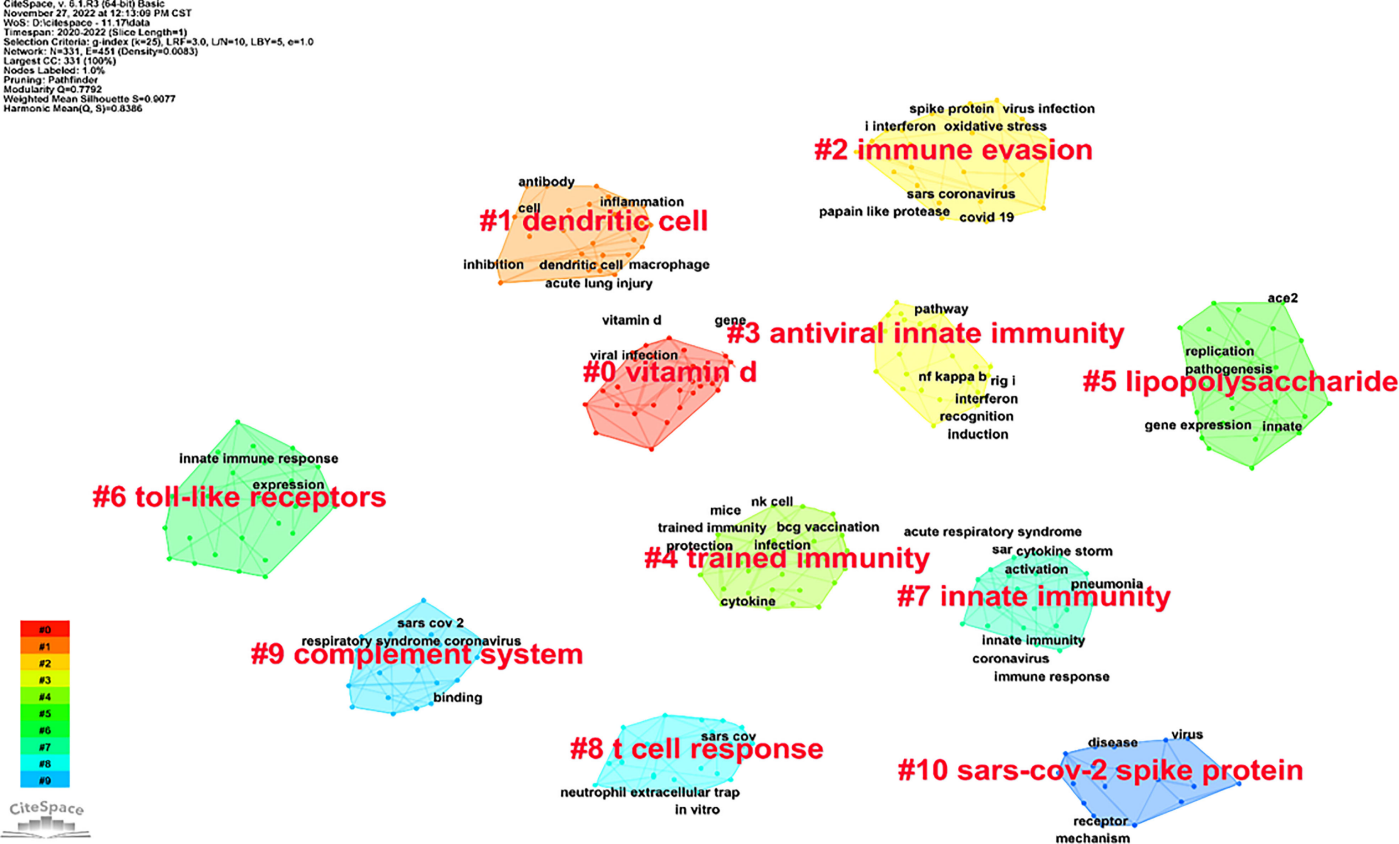
Visual map of the main clusters decided by the keywords on innate immunity in COVID-19.

**Figure 7:**
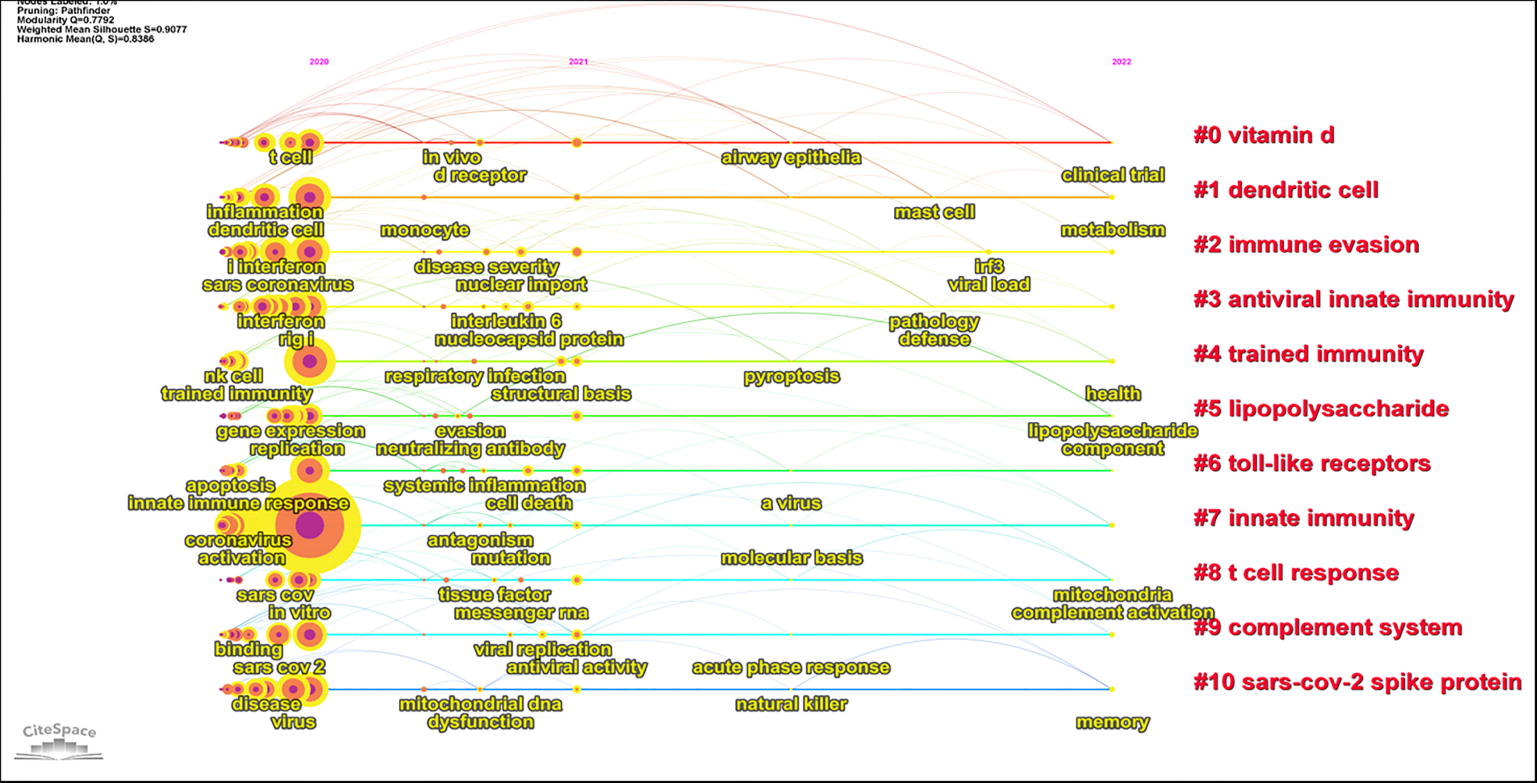
Visual map of the evolution of keywords from the main clusters.

All keywords could be classified into 11 clusters named after the highest occurring keyword (**Figure 6**; **Table 7**), and the top 3 were as follows: #0 vitamin D, #1 dendritic cell, and #2 immune evasion. The cluster labels represent the primary lines of inquiry in the area, and the terms within the same cluster were highly uniform with the modularity (0.7792) and the mean silhouette (0.9077) values greater than 0.7. The lower ID number of the cluster means a bigger size. A visual timeline of the keywords in the clusters was created to figure out the evolution of keywords. From 2020 to 2022, “inflammation,” “dendritic cell,” “interferon,” “innate immune response,” “viral replication,” “component,” and other keywords were extensively researched (**Figure 7**).

**Table 7:** The main clusters of keywords in the publications.

ID	Label	Size	Silhouette	Year	Main keyword
#0	Vitamin D	31	0.826	2020	Vitamin D, risk, viral infection
#1	Dendritic cell	29	0.91	2020	Dendritic cell, inflammation, macrophage
#2	Immune evasion	28	0.924	2020	Immune evasion, variant, chloroquine
#3	Antiviral innate immunity	26	0.864	2020	Type I interferon, nucleocapsid protein, RIG-I
#4	Trained immunity	26	0.962	2020	trained immunity, NK cell, BCG vaccination
#5	Lipopolysaccharide	26	0.95	2020	Angiotensin-converting enzyme 2, pathogenesis, lipopolysaccharide
#6	Toll-like receptors	25	0.932	2020	Innate immune response, apoptosis, toll-like receptors
#7	Innate immunity	24	0.963	2020	Innate immunity, cytokine storm, acute respiratory syndrome
#8	T cell response	22	0.948	2020	Neutrophil extracellular trap, T cell response, clinical characteristics
#9	Complement system	20	0.859	2020	Mannose binding lectin, susceptibility, complement system
#10	SARS-CoV-2 spike protein	19	0.957	2020	Receptor, mechanism, entry

### Identification of research frontiers

To provide more clues about the research frontiers in the field, the top 25 keywords with the highest burst intensity and burst year were generated using CiteSpace (**Table 8**). In 2020, studies focused on “acute respiratory syndrome,” “influenza,” and “pneumonia.” ARDS had the strongest burst, with a burst intensity of 4.7. Emerging keywords included “coronavirus disease 2019” (strength 1.89, 2021-2022), “evasion” (strength 1.76, 2021-2022), “neutralizing antibody” (strength 1.76, 2021-2022), “messenger RNA” (strength 1.76, 2021-2022), “mitochondrial DNA” (strength 1.51, 2021-2022), “respiratory infection” (strength 1.51, 2021-2022), and “toll-like receptors” (strength 1.51, 2021-2022).

**Table 8:** The top 25 keywords with the strongest citation bursts.

Keywords	Strength	Begin	End	2020-2022
acute respiratory syndrome	4.7	2020	2020	▃▂▂
influenza	3.74	2020	2020	▃▂▂
pneumonia	3.27	2020	2020	▃▂▂
mannose binding lectin	2.8	2020	2020	▃▂▂
cytokine storm	2.26	2020	2020	▃▂▂
t cell response	2.24	2020	2020	▃▂▂
clinical characteristics	2.24	2020	2020	▃▂▂
chloroquine	2.24	2020	2020	▃▂▂
sepsis	2.24	2020	2020	▃▂▂
inhibitor	2.14	2020	2020	▃▂▂
spike protein	2.12	2020	2020	▃▂▂
sars coronavirus	1.95	2020	2020	▃▂▂
convalescent plasma	1.94	2020	2020	▃▂▂
cov	1.79	2020	2020	▃▂▂
database	1.79	2020	2020	▃▂▂
angiotensin converting enzyme 2	1.5	2020	2020	▃▂▂
outbreak	1.5	2020	2020	▃▂▂
wuhan	1.5	2020	2020	▃▂▂
coronavirus disease 2019	1.89	2021	2022	▂▃▃
evasion	1.76	2021	2022	▂▃▃
neutralizing antibody	1.76	2021	2022	▂▃▃
messenger rna	1.76	2021	2022	▂▃▃
mitochondrial dna	1.51	2021	2022	▂▃▃
respiratory infection	1.51	2021	2022	▂▃▃
toll-like receptors	1.51	2021	2022	▂▃▃

The color indicate the start year and the end year of the keyword.

## Discussion

In summary, this study first summarized the current state, hotspots, and research trends on innate immunity in COVID-19 using bibliometric analysis. Our findings highlight the following: 1) the study on innate immunity in COVID-19 is a hot topic; 2) the USA and China contributed the most publications in this field; 3) collaboration between research teams and countries/regions is a prominent characteristic in this field; 4) the journal with the most publications is *Frontiers in Immunology*; and 5) studies on “messenger RNA,” “mitochondrial DNA,” and “toll-like receptors” are current and possible potential hotspots in this field.

The average number of publications in all three years was higher than 200. Moreover, the number of citations sharply increased from 2020 to 2022, demonstrating that this topic is currently hot and drawing attention from researchers. In terms of countries or regions, researchers from 87 countries or regions contributed to all publications in this field, and most of the productive countries are developed countries. Among them, the most productive country was the United States, whose Np, Nc, and H-indexes are higher than all the other countries or regions. In addition, half of the top 10 prolific institutions were from the USA. Like in other fields, possessing the most productive institutions was an important reason for the USA to contribute the most publications (30). Six of the top 10 prolific authors were from China, which partly explains why China ranked second among the top 10 productive countries.

Close collaboration between different institutions is a prominent characteristic in this field, and this was greatly facilitated by modern communication technology. Effective collaboration could, to some extent, improve the quality of publications and the academic impact of researchers and institutions. High-impact studies completed by different institutions, nations, or regions are more trustworthy (31). Especially in the context of the ongoing emergence of viral variants, researchers should improve cooperation to promote the development of the field and obtain some breakthroughs.

*Frontiers in Immunology* had the highest number of publications on innate immunity in COVID-19. On the one hand, *Frontiers in Immunology* focused on publishing studies on virology, immunology, clinical microbiology, and infection prevention, and many studies on the classification of risk factors, prevention, and treatment of COVID-19 were published in this journal (32, 33). Furthermore, more than 10 papers on innate immunity and COVID-19 have been published in *Frontiers in Immunology* in the last 3 years (34, 35). On the other hand, the relationship between innate immunity and COVID-19 is complicated and needs multidisciplinary research to determine the pathogenesis and molecular mechanisms and provide a foundation for effective treatment that matched well with the scope of *Frontiers in Immunology*.

The shifting trends of hotspots and frontiers in this field were revealed by analyzing keywords from all publications included. The top 20 most frequent keywords included “receptor,” “protein,” “cytokine storm,” “I interferon,” and “dendritic cell,” and all keywords were separated into 11 major clusters named after the highest occurring keyword in this cluster.

In cluster 0 (vitamin D cluster), vitamin D was a steroid hormone created endogenously or obtained from external dietary sources. Multiple research studies revealed that vitamin D insufficiency recently induced a wide spectrum of diseases although vitamin D research was limited to the skeletal system for a long time (36, 37). Recently, investigators found that vitamin D strengthens the immune system against viruses by various mechanisms, including the release of antiviral peptides (38). Supplementation with vitamin D may slow the disease process, and vitamin D levels are negatively correlated to the severity of COVID-19 (39, 40). Furthermore, a recent research study indicated that vitamin D may boost the host type I interferon response by increasing RIG-1/MDA-5 signaling to eradicate SARS-CoV-2 infection (41). The potential therapeutic role of vitamin D in SARS-CoV-2 infection should be further confirmed.

In cluster 1 [dendritic cell (DC) cluster], DCs bridge the innate and adaptive immune systems (42). Most DCs including plasmacytoid DCs, myeloid/conventional type 1 DCs, and myeloid/traditional type 2 DCs produced from the lymphoid primed multipotent progenitors (43). The latest studies found that reducing DCs induced type I interferon insufficiency and delayed adaptive immune activation, which would impair the ability to fight SARS-CoV-2 (44, 45). However, the function of DCs in SARS-CoV-2 infection has not been fully explored.

In cluster 2 (immune evasion cluster), immune evasion was mainly induced by SARS-CoV-2 variants, which significantly increased the difficulty in stopping the virus spread and triggered a more transmissible wave of infections worldwide during the COVID-19 pandemic (46). Compared with the original strain, the infectivity and immune evasion of some variants of SARS-CoV-2, such as Alpha, Beta, Delta, and Omicron, were both higher (47–49). Notably, the Omicron variant, which first emerged at the end of 2021, has become the most prevalent variant and has posed challenges in controlling the outbreak (50). Structural analysis of the spike protein from the Omicron variant using cryoelectron microscopy revealed that the altered amino acid viral structure improved viral adherence and significantly improved immune evasion to avoid recognition by the immune system (51).

Combined with the visual timeline and citation burst of keywords, studies on “messenger RNA,” “mitochondrial DNA,” and “toll-like receptor” were found to be possible directions for current and future research in this field.

Messenger RNA, which transports genetic information from the DNA to the ribosome for protein synthesis, was highlighted by researchers in 1961 (52). The clinical applications of messenger RNA were increasing with the development of nucleoside-modified and lipid nanoparticle-facilitated delivery technologies (53). The first vaccine for COVID-19 added to the WHO emergency use list was the messenger RNA vaccine (BNT162b2) produced by Pfizer and BioNTech, which reflects the rapid deployment of messenger RNA vaccines and the vital role of studying messenger RNA in this field (54).

The mitochondria affect host cell homeostasis and metabolism and are crucial for the activation of cell death and immunological signaling (55). The release of mitochondrial DNA may cause a severe innate immune response by pattern recognition receptors (56). Mitochondrial DNA interacts with cyclic GMP-AMP synthase-stimulator of interferon genes, NOD-like receptor protein inflammasomes, and melanoma inflammasomes, which would induce invasive cytokine storms during SARS-CoV-2 infection and has a negative clinical impact (57). Further research on the mechanism of mitochondrial DNA in COVID-19 may provide a foundation for a new therapeutic target.

TLRs, a kind of type I transmembrane proteins, consist of three structural domains (58). TLRs activate the NF-kB and interferon regulatory factors, which further trigger the production of inflammatory cytokines and interferons by binding to ligands and intracellular immune signaling through myeloid differentiation factor 88 and the toll/interleukin-1 receptor domain (59, 60). The SARS-CoV-2 spike protein has been reported to bind to TLR1/2 or TLR2/6, which further caused an inflammatory response by activating the NF-kB signaling pathways and mitogen-activated protein kinases in macrophages, monocytes, and human lung epithelial cells (61). Additionally, in COVID-19 patients, the level of TLR2 was positively related to disease severity (62). Another study showed that the spike protein could bind to TLR4 which caused the release of inflammatory cytokines like tumor necrosis factor-α and IL-6 by the NF-kB signaling pathway (63). Although moderate TLR activation accelerates virus elimination, excessive TLR activation causes tissue damage and even death (64). The above data implied that TLR2 or TLR4 is a key receptor in establishing inflammatory response. Therefore, further study on TLRs and spike proteins will possibly assist in elucidating the pathogenesis, immune evasion, and treatment of COVID-19.

### Limitations

A major limitation of this study is that the collection of publications was limited to the WoSCC database, and the results may differ when other databases are adapted. Second, only author keywords were analyzed, and the results may be different based on index keywords or keywords plus. Third, the analysis of keywords may be affected by personal preference of using keywords.

## Conclusions

Research on innate immunity in COVID-19 is a hot topic and deserves global attention. The USA is the leading country with the highest number of publications on innate immunity in COVID-19, followed by China. Collaboration between different institutions and researchers is a significant characteristic of this field. The *Frontiers in Immunology* journal has the highest number of publications in this field. “Messenger RNA,” “mitochondrial DNA,” and “toll-like receptors” are the three current research hotspots and possible potential directions for future research.

## Data availability statement

The original contributions presented in the study are included in the article/supplementary material. Further inquiries can be directed to the corresponding authors.

## Author contributions

This study was designed by PL, Y-LL and SX. All data were enrolled by SX, J-HX and PL. All data were analyzed by PL, SX, J-HX, H-ZZ and Y-MZ. PL, SX and Y-LL drew the figures. PL and SX drafted the manuscript. PL, J-HX and Y-LL revised the final version of the manuscript. All authors contributed to the article and approved the submitted version.
